# Risk of Type 2 Diabetes, MASLD and Cardiovascular Disease in People Living With Polycystic Ovary Syndrome

**DOI:** 10.1210/clinem/dgae481

**Published:** 2024-07-11

**Authors:** Alex E Henney, Conor S Gillespie, Jonathan Y M Lai, Pieta Schofield, David R Riley, Rishi Caleyachetty, Thomas M Barber, Alexander D Miras, Laurence J Dobbie, David M Hughes, Uazman Alam, Theresa J Hydes, Daniel J Cuthbertson

**Affiliations:** Department of Cardiovascular & Metabolic Medicine, University of Liverpool, Liverpool L69 7ZX, UK; Metabolism & Nutrition Research Group, Liverpool University Hospitals NHS Foundation Trust, Liverpool, Merseyside L9 7AL, UK; Liverpool Centre for Cardiovascular Sciences, University of Liverpool and Liverpool University Hospitals NHS Foundation Trust, Liverpool, Merseyside L69 7ZX, UK; Department of Clinical Neurosciences, University of Cambridge, Cambridge CB2 3PT, UK; Metabolism & Nutrition Research Group, Liverpool University Hospitals NHS Foundation Trust, Liverpool, Merseyside L9 7AL, UK; Department of Public Health, Policy & Systems, University of Liverpool, Liverpool, Merseyside L69 7ZX, UK; Department of Cardiovascular & Metabolic Medicine, University of Liverpool, Liverpool L69 7ZX, UK; Metabolism & Nutrition Research Group, Liverpool University Hospitals NHS Foundation Trust, Liverpool, Merseyside L9 7AL, UK; Warwick Medical School, University of Warwick, Coventry CV4 7AL, UK; Warwick Medical School, University of Warwick, Coventry CV4 7AL, UK; Department of Metabolism, Digestion and Reproduction, Imperial College London, Hammersmith Hospital, London SW7 2AZ, UK; Department of Diabetes and Endocrinology, Guy's and St Thomas’ NHS Foundation Trust, London SE1 7EH, UK; Institute of Population Health, University of Liverpool, Liverpool L69 7ZX, UK; Department of Cardiovascular & Metabolic Medicine, University of Liverpool, Liverpool L69 7ZX, UK; Metabolism & Nutrition Research Group, Liverpool University Hospitals NHS Foundation Trust, Liverpool, Merseyside L9 7AL, UK; Liverpool Centre for Cardiovascular Sciences, University of Liverpool and Liverpool University Hospitals NHS Foundation Trust, Liverpool, Merseyside L69 7ZX, UK; Department of Cardiovascular & Metabolic Medicine, University of Liverpool, Liverpool L69 7ZX, UK; Metabolism & Nutrition Research Group, Liverpool University Hospitals NHS Foundation Trust, Liverpool, Merseyside L9 7AL, UK; Department of Gastroenterology and Hepatology, Liverpool University Hospitals NHS Foundation Trust, Liverpool, Merseyside L9 7AL, UK; Department of Cardiovascular & Metabolic Medicine, University of Liverpool, Liverpool L69 7ZX, UK; Metabolism & Nutrition Research Group, Liverpool University Hospitals NHS Foundation Trust, Liverpool, Merseyside L9 7AL, UK; Liverpool Centre for Cardiovascular Sciences, University of Liverpool and Liverpool University Hospitals NHS Foundation Trust, Liverpool, Merseyside L69 7ZX, UK

**Keywords:** polycystic ovary syndrome, type 2 diabetes, metabolic dysfunction-associated steatotic liver disease, cardiovascular disease, hormone-dependent cancers

## Abstract

**Background:**

Polycystic ovary syndrome (PCOS) is associated with adverse clinical outcomes that may differ according to PCOS phenotype.

**Methods:**

Using UK Biobank data, we compared the incidence of type 2 diabetes (T2D), metabolic dysfunction associated steatotic liver disease, cardiovascular disease (CVD), hormone-dependent cancers, and dementia between PCOS participants and age- and body mass index-matched controls. We also compared multiorgan (liver, cardiac, and brain) magnetic resonance imaging (MRI) data and examined the impact of PCOS phenotype (hyperandrogenic and normoandrogenic) on these outcomes.

**Results:**

We included 1008 women with PCOS (defined by diagnostic codes, self-reported diagnoses, or clinical/biochemical features of hyperandrogenism and a/oligoCmenorrhoea) and 5017 matched controls (5:1 ratio); median age, 61 years, body mass index, 28.4 kg/m². Adjusted Cox proportional hazard modeling demonstrated PCOS participants had greater incident T2D [hazard ratio (HR) 1.47; 95% confidence interval (CI), 1.11-1.95] and all-cause CVD (1.76; 1.35-2.30). No between-group differences existed for cancers or dementia. Liver MRI confirmed more PCOS participants had hepatic steatosis (proton density fat fraction >5.5%: 35.9 vs 23.9%; *P* = .02) and higher fibroinflammation (corrected T1 721.4 vs 701.5 ms; *P* = <.01) vs controls. No between-group difference existed for cardiac (biventricular/atrial structure and function) or brain (grey and white matter volumes) imaging. Normoandrogenic (but not hyperandrogenic) PCOS participants had greater incident all-cause CVD (1.82; 1.29-2.56) while hyperandrogenic (but not normoandrogenic) PCOS participants were more likely to have hepatic steatosis (8.96 vs 6.04 vs 5.23%; *P* = .03) with greater fibroinflammation (776.3 vs 707.7 vs 701.9 ms; *P*=<.01).

**Conclusion:**

Cardiometabolic disease may be increased in PCOS patients with a disease phenotype-specific pattern.

Polycystic ovary syndrome (PCOS) is the most common endocrine disorder in women of reproductive age, affecting 4% to 21% of women dependent upon diagnostic criteria, clinical setting, and ethnicity (1). The Rotterdam criteria are the most widely accepted (2), but closer subtyping of PCOS according to phenotype, specifically according to serum androgen concentrations, may help more accurate risk stratification (3). Approximately 50% of PCOS patients are living with obesity, with excess childhood and adolescent adiposity influencing the pathophysiology of adult PCOS (4).

Observational data suggests PCOS increases cardiometabolic disease risk, including type 2 diabetes (T2D), metabolic dysfunction associated steatotic liver disease (MASLD), obstructive sleep apnoea (OSA), cardiovascular disease (CVD), certain gynecological malignancies, most notably endometrial cancer, and cognitive decline (3, 5-17). The pathophysiological processes driving these associations are unclear but may partly be explained by concomitant cardiovascular risk factors in people with PCOS resulting from obesity (18). Despite this, PCOS is characterized by intrinsic insulin resistance, independent of body weight (19), perhaps related to unfavorable body composition with greater liver fat deposition (3), and hence these intrinsic PCOS characteristics may amplify adverse clinical outcomes.

To date, limited prospective studies in women with PCOS analyze longer-term health consequences, extending into the postmenopausal period, contributing to heterogeneity in study findings (5, 6, 11, 20). Additionally, in an era of precision medicine, disease phenotyping can differentiate between PCOS phenotypes, but no large studies have explored disease associations while attempting to provide mechanistic insights.

We aimed to determine whether (1) people with PCOS are at increased risk of metabolic disease (T2D, MASLD, and OSA), CVD, related cancers, or dementia, independent of traditional risk factors; (2) multiparametric magnetic resonance imaging (MRI) findings could explain putative disease associations; and (3) these risks differ according to PCOS phenotype.

## Methods

### Study Population

The UK Biobank (UKBB) is a nationwide, longitudinal, biomedical database containing in-depth health information (21). Recruitment of >500 000 participants, aged 40 to 69, occurred between 2006 and 2010 by postal invitation. Approximately 60 000 have had multiorgan MRI performed. Participants attended 1 of 22 assessment centers in England, Scotland, or Wales, where they completed demographic, medical history, and lifestyle questionnaires during a baseline assessment visit. Additionally, self-reported doctor-diagnosed medical conditions were verified and coded during a face-to-face interview, and a physical examination was performed, with collection of blood and urine samples. All participants consented to linkage of their electronic health records to enable prospective data collection (death and cancer records held by the Office for National Statistics and the Registrar General's Office; hospital records held by the Department of Health's Hospital Episode Statistics; Scottish Morbidity Records). At the time of analysis, mortality and hospital admission data were available to April 2023. Ethics approval was granted by the North West Multi-Centre Research Ethics Committee. Permission to access UKBB data was approved under project 74170.

### Inclusion Criteria

**Figure 1.**
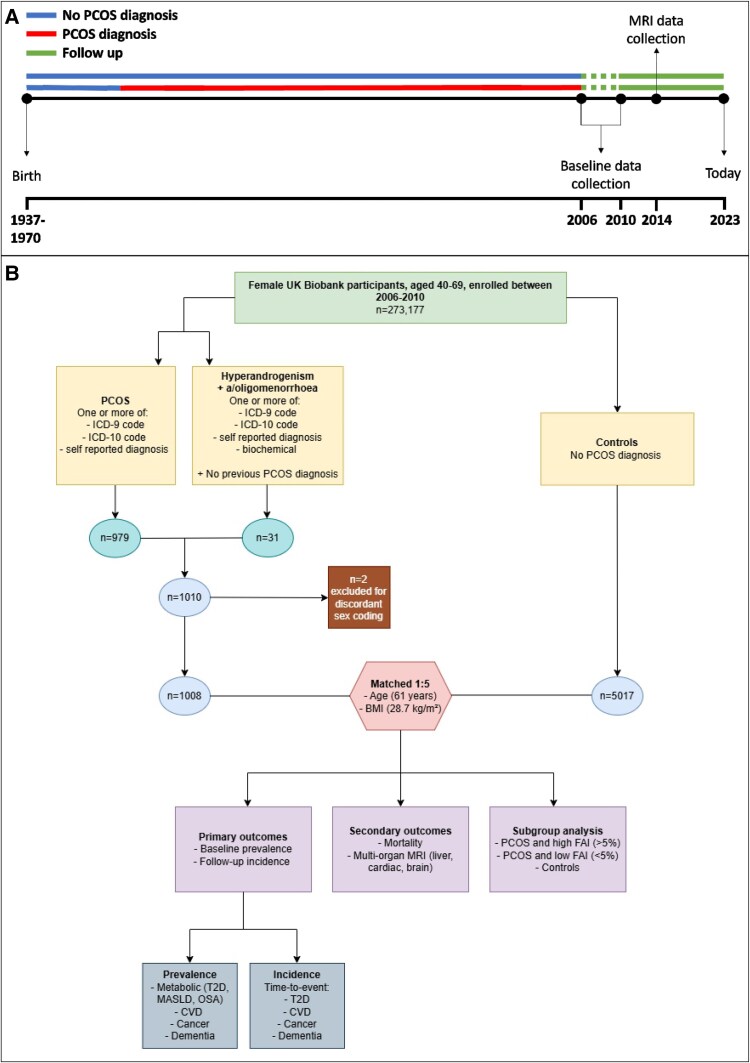
Study overview: (A) timeline representing the historical PCOS diagnosis, baseline data collection point, and follow-up period. (B) Attrition chart for identification of the PCOS cohort. Abbreviations: BMI, body mass index; CVD, cardiovascular disease; FAI, free androgen index; ICD, International Classification of Disease; MASLD, metabolic dysfunction associated steatotic liver disease; MRI, magnetic resonance imaging; OSA, obstructive sleep apnea; PCOS, polycystic ovary syndrome; T2D, type 2 diabetes.

We identified all participants within the UKBB who had a previous PCOS diagnosis at their baseline assessment (Fig. 1). PCOS was defined using an International Classification of Diseases, Ninth/Tenth Revision (ICD-9/10) code or a (doctor-verified) self-reported diagnosis. We additionally included people with clinical codes and/or biochemical features of both hyperandrogenism and secondary amenorrhoea/oligomenorrhoea, without other coding for PCOS, in line with Rotterdam criteria (2) [Supplemental Table 1 (22)]. A control group, not known to have PCOS, was matched for age and body mass index (BMI) in a 5:1 ratio with cases. The index date, acting as a marker for baseline characteristics of cases and controls, was set as the first date each participant attended a UKBB assessment center.

### Exclusion Criteria

Participants were excluded if they had a diagnosis of PCOS with a discordant self-reported sex or if they withdrew consent for the use of their data. Moreover, we excluded any control participant if they had discordant coding for sex or coding for transsexualism/genderism (ICD-9 code 302.5, ICD-10 code F64). Additional exclusions were made dependent on the outcomes described next.

### Primary Outcomes

Primary outcomes were measured at baseline and follow-up. First, baseline prevalence of (1) metabolic disease (T2D, MASLD, OSA); (2) CVD: all-cause CVD and individual CVD components [heart failure, acute coronary syndrome, atrial fibrillation, cardiomyopathy, cerebrovascular accident (ischaemic or hemorrhagic stroke or transient ischemic event), or peripheral arterial disease]; (3) cancer: hormone-dependent (breast, ovary, endometrial, colorectal) and hepatobiliary cancers (liver and pancreas, excluding cancers of neuroendocrine origin); and (4) dementia (Alzheimer's disease, vascular or frontotemporal dementia, excluding dementia secondary to Creutzfeldt-Jakob, Parkinson's, Huntington's, or human immunodeficiency virus disease) was assessed [Supplemental Tables 2-8 (22)].

Second, follow-up data was analyzed to determine the time-to-incident T2D, CVD (fatal or nonfatal), cancer, and dementia. Any participant who reported the outcome of interest at baseline was excluded from the prospective analysis.

All outcomes were ascertained by ICD-9/10 or self-reported, codes [Supplemental Tables 2-8 (22)]. T2D was additionally identified through medication used to treat T2D or a glycated hemoglobin (HbA1c) ≥ 48 mmol, excluding other causes of diabetes [Supplemental Table 2 (22)]. PCOS participants using metformin, but not meeting other T2D criteria, were not defined as having T2D. MASLD was additionally identified through a Hepatic Steatosis Index score >36 (23) [Supplemental Table 9 (22)]. Participants with MASLD at baseline must have been free of other causes of chronic liver disease. For all-cause CVD, if a participant experienced multiple cardiovascular events, the first was included.

### Secondary Outcomes

Time-to all-cause mortality and multiorgan MRI data (liver, cardiac and brain) were assessed [Supplemental Table 10 (22)].

#### Multiorgan imaging

Perspectum provided abdominal MRI data [Supplemental Fig. 1 (22)]. Cardiovascular magnetic resonance data enables objective assessment of cardiac structure and function. Brain imaging uses preprocessed T1/T2 structural brain image data generated by an image-processing pipeline developed and run on behalf of UKBB. A detailed description on the imaging technique is presented in the Supplemental Material Additional Reading (22).

### Subgroup Analysis

We stratified PCOS participants based on androgen level into 2 phenotypes: hyperandrogenic and normoandrogenic. Hyperandrogenism was defined as a Free Androgen Index (FAI) > 5%; 100 × [testosterone concentration (nmol/liter)/SHBG concentration (nmol/liter)] (24). The same primary and secondary outcomes were compared. Participants in the main study were excluded from subgroup analysis if insufficient data was available to calculate a FAI.

### Statistical Analysis

Baseline characteristics are presented as percentages, and continuous data are presented using mean ± SD (parametric data) or median with interquartile range (nonparametric data).

Crude rates of progression to each primary outcome are presented, and time to each end point was analyzed using univariate and multivariable Cox proportional hazards modeling (CPHMs). Hazard ratios (HR) with 95% confidence intervals (CIs) are presented following CPHM. Missing data was dealt with by complete case analysis. Non-cases were censored at the date of loss to follow-up, date of death, or end of follow-up. The CPHM models included the following covariates: model 1 adjusted for age, ethnicity, and Index of Multiple Deprivation [7 domains of deprivation are considered and weighted as follows: Income (22.5%), Employment (22.5%), Education (13.5%), Health (13.5%), Crime (9.3%), Barriers to Housing and Services (9.3%), and Living Environment (9.3%)]. Model 2 additionally adjusted for smoking and alcohol, and Model 3 additionally adjusted for HbA1c, all-cause CVD, dyslipidemia, and hypertension [Supplemental Material (22)]. The secondary outcome of all-cause mortality, and subgrouped analysis by PCOS phenotype, were also analyzed using CPHMs. Model 4 was included for subgroup analysis to adjust for BMI, which was no longer matched between subgroups.

Sensitivity analysis was performed for all CPHM results through the calculation of E-values, representing the minimum strength of association on the HR scale that an unmeasured confounder would need to have with both PCOS and the outcome, conditional on the measured confounders, to explain away the observed association; HR+√[HR × (HR-1)] (25).

Liver, cardiac, and brain imaging data were compared between participants with PCOS and controls using *t*-test, whereas for subgroup analysis, means were compared using analysis of covariance, adjusting for BMI. Statistical significance was taken as *P* =< .05.

Data was analyzed using RStudio (v4.3.2). The Strengthening the Reporting of Observational Studies in Epidemiology guidelines were followed in the reporting of this prospective cohort study.

## Results

### Identification of a Cohort of Individuals With PCOS Within the UKBB

Of the 502 516 volunteers with UKBB data, 6025 participants were analyzed in this study: 1008 had a diagnosis of PCOS [Supplemental Table 1 (22)], with a control sample of 5017 matched for age and BMI. Two patients with PCOS were excluded for discordant sex identification (Fig. 1). Baseline demographics are presented in Table 1.

**Table 1.** Baseline characteristics and prior comorbidities for patients with PCOS and controls

	Total	PCOS	Control	SMD
n	6025	1008	5017	
Demographic variables				
Age, years	Median	61	61	.00
Ethnicity				
White	%	86.1	87	.03
Non-White	%	13.9	13	
Index of Multiple Deprivation	Mean (SD)	19.1 (14.0)	20.6 (15.2)	.10
Fertility				
Live childbirths, n	Mean (SD)	1.2 (1.2)	1.6 (1.3)	.31
Lifestyle variables, %				
Alcohol intake				
High (3 or more times a week)		27.0	30.6	.08
Moderate (once a month to twice a week)		41.7	42.0	.01
Low (never or special occasions)		31.3	27.4	.09
Smoking status				
Current smoker		8.6	11.6	.10
Physical activity level				
Meets physical activity guidelines		8	8.9	.03
Cardiometabolic risk markers				
Dyslipidaemia	%	7.2	5	.09
Blood pressure				
Hypertension	%	26.6	19	.18
Diastolic blood pressure, mmHg	Median (IQR)	81 (74-88)	81 (74-89)	.01
Systolic blood pressure, mmHg	Median (IQR)	130 (119-143)	130 (119-143)	.02
Biochemistry, median (IQR)				
Liver enzymes, IU/L				
ALT		17.7 (13.5-24.2)	16.8 (13.3-22.6)	.10
AST		22 (18.9-26.2)	21.7 (18.8-25.3)	.05
GGT		21.4 (15.6-34.3)	21.3 (15.8-31.5)	.11
Endocrine biochemistry				
Oestradiol, nmol/L		380 (258.2-633.3)	418 (273.3-631.1)	.04
SHBG, nmol/L		47.6 (32.1-72.1)	52.3 (36-73.9)	.07
Testosterone, nmol/L		1.2 (.9-1.6)	1.1 (.8-1.5)	.15
IGF-1, IU/L		21.9 (17.6-26.2)	21.9 (17.9-25.8)	<.01
Lipid profile, mmol/L				
LDL		1.7 (1.4-2)	1.6 (1.4-1.9)	.13
HDL		1.3 (1.1-1.5)	1.3 (1.1-1.6)	.07
Total cholesterol		4.6 (3.9-5.1)	4.4 (3.9-5)	.10
Triglycerides		1.1 (0.8-1.6)	1 (0.8-1.4)	.24
Prevalence of comorbid disease				
Overweight/obesity				
BMI, kg/m²	Median (IQR)	28.7 (24.6-34.8)	28.7 (24.6-34.8)	.00
Overweight, BMI 25-30 kg/m²	%	29.5	29.9	.01
Obesity, BMI >30 kg/m²	%	42.8	42.5	.01
Waist circumference, cm	Median (IQR)	88 (78-102)	87 (78-101)	.06
Type 2 diabetes				
Type 2 diabetes	%	7.3	4	.14
Prediabetes	%	4.1	3.5	.03
HbA1c, mmol/mol	Median (IQR)	34.7 (31.9-37.6)	34.2 (31.7-37)	.13
HbA1c in those with diabetes, mmol/mol	Median (IQR)	50.8 (42.2-66.6)	52.9 (47.1-62.8)	.05
MASLD, %				
MASLD		58.7	54.8	.08
Steatosis indices				
HSI > 36		58.7	54.8	.08
Fibrosis indices				
FIB-4 > 2.67		1.6	1.8	.02
NFS > .675		3	5.9	.14
OSA, %		0.6	0.4	.09
All-cause cardiovascular disease, %		3.2	2.3	.06
All-cause hormone-dependant cancers, %		3.3	2.8	.03
Hepatobiliary cancers, %				
Liver		0.1	0	.2
Pancreas		0	<.01	0.02
Psychiatric disorder, %				
Depression and/or anxiety		16.2	11.3	.14
All-cause dementia		0	<.1	.02

Meeting physical activity guidelines was defined as surpassing >225 minutes per week of moderate activity or 75 minutes of vigorous activity.

Abbreviations: ALT, alanine transaminase; AST, aspartate transaminase; BMI, body mass index; FIB-4, fibrosis index-4; GGT, gamma-glutamyl transferase; HbA1c, glycated hemoglobin; HDL, high-density lipoprotein; HSI, Hepatic Steatosis Index; IGF-1, insulin-like growth factor-1; IQR, interquartile range; LDL, low-density lipoprotein; MASLD, metabolic dysfunction associated steatotic liver disease; NFS, nonalcoholic fatty liver disease fibrosis score; OSA, obstructive sleep apnea; PCOS, polycystic ovary syndrome; SMD, standardized mean difference.

Cases and controls had a median age of 61 years and BMI of 28.7 kg/m². At baseline, participants with PCOS had a lower level of (socioeconomic) deprivation, lower rates of smoking, and a lower fertility rate. Further, they had higher serum concentrations of liver transaminases (alanine transaminase and gamma-glutamyl transferase), lipid profile (low-density lipoprotein and total cholesterol), HbA1c, and free testosterone. There was no difference between participants with PCOS and controls with respect to alcohol consumption, physical activity levels, or ethnic composition.

### Prevalent Cardiometabolic Disease, Cancer, and Dementia

The prevalence of T2D (7.3 vs 4%), hypertension (26.6 vs 19.0%), hepatocellular carcinoma (0.1 vs 0.0%), and depression/anxiety (16.2 vs 11.3%) was higher in participants with PCOS (vs controls). There was no difference in respect to the prevalence of hormone-dependent or pancreatic cancer, other CVD, or dementia (Table 1).

### Incident Cardiometabolic Disease, Cancer, and Dementia

All CPHM results are presented in Table 2, while a forest plot and cumulative incidence curves are presented in Supplemental Fig. 2 to 4 (22). E-values are presented in Supplemental Table 11 (22).

**Table 2.** Number, crude rates, and associated HRs for incident metabolic, cardiovascular, cancer, and dementia events in women with PCOS and matched controls

Outcome	Group	No.	Events	Mean follow up, years (SD)	Incidence rate (per 1000 persons)	Univariate model	Multivariable model 1	Multivariable model 2	Multivariable model 3
Type 2 diabetes, HR (95% CI)									
Type 2 diabetes	Controls	4134	227	13.6 (2.1)	54.9	1.00	1.00	1.00	1.00
	PCOS	847	69	13.5 (2.3)	81.5	**1.59 (1.21-2.08)**	**1.65 (1.25-2.19)**	**1.62 (1.22-2.15)**	**1.47 (1.11-1.95)**
Cardiovascular disease, HR (95% CI)									
Total	Controls	4883	269	13.8 (1.8)	55.1	1.00	1.00	1.00	1.00
	PCOS	976	89	13.6 (2.2)	91.2	**1.69 (1.33-2.15)**	**1.87 (1.46-2.40)**	**1.86 (1.44-2.39)**	**1.76 (1.35-2.30)**
Heart failure	Controls	5008	69	13.9 (1.7)	13.8	1.00	1.00	1.00	1.00
	PCOS	1006	25	13.8 (1.7)	24.9	**1.82 (1.15-2.88)**	**2.22 (1.38-3.57)**	**2.14 (1.33-3.46)**	**1.83 (1.08-3.12)**
Acute coronary syndrome	Controls	4982	69	13.9 (1.7)	13.9	1.00	1.00	1.00	1.00
	PCOS	1001	24	13.9 (1.5)	24.0	**1.67 (1.03-2.71)**	**2.00 (1.22-3.28)**	**2.00 (1.22-3.28)**	**2.02 (1.21-3.37)**
Cardiomyopathy	Controls	5011	9	13.9 (1.7)	1.8	1.00	1.00	1.00	1.00
	PCOS	1007	6	13.9 (1.5)	6.0	2.77 (0.93-8.27)	**3.37 (1.07-10.63)**	**3.98 (1.20-13.17)**	**4.40 (1.25-15.51)**
Atrial fibrillation	Controls	5003	97	13.8 (1.7)	19.4	1.00	1.00	1.00	1.00
	PCOS	1003	31	13.9 (1.6)	30.9	**1.61 (1.07-2.41)**	**1.80 (1.19-2.73)**	**1.82 (1.20-2.77)**	**1.75 (1.12-2.72)**
Cerebrovascular accident	Controls	4959	69	13.8 (1.8)	13.9	1.00	1.00	1.00	1.00
	PCOS	997	23	13.8 (1.7)	23.1	**1.69 (1.05-2.71)**	**1.70 (1.03-2.79)**	**1.69 (1.02-2.78)**	1.69 (0.99-2.91)
Peripheral arterial disease	Controls	4998	32	13.9 (1.7)	6.4	1.00	1.00	1.00	1.00
	PCOS	1001	10	13.9 (1.6)	10.0	1.57 (0.77-3.20)	1.63 (0.74-3.61)	1.76 (0.79-3.90)	1.30 (0.55-3.06)
Cancer, HR (95% CI)									
Hormone-dependent cancer									
Total	Controls	4874	231	13.7 (2.2)	47.4	1.00	1.00	1.00	1.00
	PCOS	975	61	13.5 (2.3)	62.6	**1.37 (1.03-1.82)**	1.32 (0.97-1.78)	1.33 (0.98-1.81)	1.29 (0.94-1.77)
Breast	Controls	4917	172	13.7 (2.1)	35.0	1.00	1.00	1.00	1.00
	PCOS	988	47	13.6 (2.2)	47.6	**1.44 (1.04-1.98)**	1.38 (0.98-1.94)	**1.41 (1.00-1.99)**	1.31 (0.91-1.88)
Ovary	Controls	5002	28	13.9 (1.7)	5.6	1.00	1.00	1.00	1.00
	PCOS	1002	5	13.8 (1.7)	5.0	0.89 (0.34-2.31)	0.92 (0.32-2.68)	0.91 (0.31-2.66)	0.88 (0.30-2.59)
Endometrium	Controls	4998	6	13.9 (1.6)	1.2	1.00	1.00	1.00	1.00
	PCOS	1002	0	13.9 (1.5)	NA	0.04 (0.00-412)	0. (0.00-NA)	0.00 (0.00-NA)	0.00 (0.00-NA)
Colorectal	Controls	5004	34	13.9 (1.7)	6.8	1.00	1.00	1.00	1.00
	PCOS	1004	13	13.8 (1.6)	12.9	**1.96 (1.03-3.73)**	1.68 (0.82-3.44)	1.67 (0.82-3.43)	1.77 (0.86-3.64)
Hepatobiliary cancer									
Liver	Controls	5017	0	14.1 (1.0)	0	1.00	1.00	1.00	1.00
	PCOS	1007	0	14.1 (1.0)	0	NA	NA	NA	NA
Pancreas	Controls	5016	11	14.1 (1.1)	2.2	1.00	1.00	1.00	1.00
	PCOS	1008	0	14.1 (1.0)	0	0.04 (0.00-35.59)	0.00 (0.00-NA)	0.00 (0.00-NA)	0.00 (0.00-NA)
Dementia, HR (95% CI)									
Dementia	Controls	5016	26	13.9 (1.7)	5.2	1.00	1.00	1.00	1.00
	PCOS	1008	6	13.9 (1.5)	6.0	1.16 (0.48-2.81)	1.16 (0.44-3.07)	1.11 (0.42-2.95)	0.96 (0.32-2.89)
All-cause mortality, HR (95% CI)									
Mortality	Controls	5017	184	13.9 (1.6)	36.7	1.00	1.00	1.00	1.00
	PCOS	1008	46	13.9 (1.5)	45.6	1.23 (0.91-1.73)	1.33 (0.94-1.90)	1.32 (0.93-1.89)	1.13 (0.77-1.66)

Model 1: age, Index of Multiple Deprivation, ethnicity; model 2: age, Index of Multiple Deprivation, ethnicity, smoking, alcohol; model 3: age, Index of Multiple Deprivation, ethnicity, smoking, alcohol, dyslipidemia, hypertension, cardiovascular disease, and/or glycated hemoglobin (dependent upon outcome). Results in bold carry significance.

Abbreviations: CI, confidence interval; HR, hazard ratio; PCOS, polycystic ovary syndrome; NA, not available.

#### T2D

The incidence rate per 1000 person-years was higher in participants with PCOS than controls (81.5 vs 54.9). Univariate analysis revealed a significant association between PCOS and T2D incidence (HR 1.59; 95% CI, 1.21-2.08), which remained after full multivariable adjustment (HR 1.47; 95% CI, 1.11-1.95).

#### CVD

The all-cause CVD incidence rate per 1000 person-years was higher in participants with PCOS than controls (91.2 vs 55.1). Univariate analysis revealed a significant association between PCOS and all-cause CVD incidence (HR 1.69; 95% CI, 1.33-2.15), which remained after full multivariable adjustment (HR 1.76; 95% CI, 1.35-2.30).

#### Cancer

The incidence rate per 1000 person-years was higher in participants with PCOS than controls for all-cause hormone-dependent cancer (62.6 vs 47.4). Univariate analysis revealed a significant association between PCOS and incident all-cause hormone-dependent cancer (HR 1.37; 95% CI, 1.03-1.82), which disappeared after full multivariable adjustment.

#### Dementia

The all-cause dementia incidence rate per 1000 person-years was higher in participants with PCOS than controls (6.0 vs 5.2). Univariate analysis revealed no significant association between PCOS and all-cause dementia (HR 1.16; 95% CI, 0.48-2.81), remaining insignificant after full multivariable adjustment (HR 0.96; 95% CI, 0.32-2.89).

#### All-cause mortality

The all-cause mortality rate per 1000 person-years was higher in participants with PCOS than controls (45.6 vs 36.7). Univariate analysis revealed no significant association between PCOS and all-cause mortality (HR 1.23, 95% CI, 0.91-1.73), remaining insignificant after full multivariable adjustment (HR 1.13; 95% CI, 0.77-1.66) (Table 2).

### Multiorgan Imaging

Results from all imaging parameters are presented in Table 3.

**Table 3.** Number of participants undergoing multiorgan (liver, heart, and brain) imaging, and the mean values of respective imaging parameters for participants with PCOS and controls

	PCOS	Control	*P*
Liver, mean (SD)	117	402	
PDFF, %	6.2 (6.4)	5.2 (5.8)	.1
PDFF above normal (>5.5), %	**35.9**	**23.9**	.**02**
Liver iron	1.2 (0.2)	1.2 (0.2)	.09
Liver iron corrected T1, ms	**721.4** (**63.9)**	**701.5** (**58.4)**	**<**.**01**
Liver iron corrected T1 above normal (>800 ms), %	12.7	7.7	.11
Body composition, mean (SD)	112	395	
Abdominal subcutaneous adipose tissue volume, L	9.5 (4.4)	9.7 (4.6)	.64
Abdominal visceral adipose tissue volume, L	3.1 (1.8)	3.2 (1.8)	.58
Cardiac, mean (SD)	106	384	
LV EF, %	59.8 (6.0)	60.8 (5.5)	.1
LV SV, ml	81.7 (16.3)	83.8 (15.8)	.22
LV longitudinal strain, %	−19.0 (2.9)	−19.1 (2.7)	.7
LV mass, g	75.0 (15.7)	75.6 (14.9)	.70
LA EF, %	61.8 (9.4)	62.1 (8.1)	.7
LA SV, ml	43.4 (11.4)	44.4 (11.4)	.4
LA max volume, ml	71.6 (20.2)	72.5 (20.2)	.7
Ascending aorta distensibility, 10^−3^mmHg^−1^	2.2 (1.4)	2.3 (1.6)	.7
Brain, mean (SD)	130	469	
Grey matter volume, mm^3^	610707.1 (52530.4)	608297.7 (49781.1)	.6
White matter volume, mm^3^	519737.1 (49979.4)	520166.5 (50608.5)	.9
White matter hyperintensities total volume, mm^3^	2902.2 (3270.6)	3157.5 (4586.1)	.6

Abnormal PDFF taken at >5.5% and abnormal corrected T1 taken at >800 ms. Results in bold carry significance.

Abbreviations: EF, ejection fraction; LA, left atrial; LV, left ventricular; PCOS, polycystic ovary syndrome; PDFF, proton density fat fraction; SV, stroke volume.

#### Liver/adipose tissue depot volumes

Of participants with PCOS and controls, 11.6% and 8.0%, respectively, underwent abdominal MRI, whereas 11.1 and 7.9%, respectively, underwent assessment of body composition. More PCOS participants had a proton density fat fraction (PDFF) exceeding 5.5% than controls (35.9 vs 23.9%; *P* = 0.02), while those with PCOS also had significantly higher liver corrected T1 (cT1) [721.4 (63.9) vs 701.5 (58.4); *P* = <.01)] [Supplemental Fig. 5 (22)]. No significant differences existed between groups with respect to abdominal subcutaneous or visceral adipose tissue volumes.

#### Cardiac

Of participants with PCOS and controls, 10.5% and 7.7%, respectively, underwent cardiovascular magnetic resonance. No significant differences existed between groups in respect to any biatrial or biventricular, structural, or functional parameters.

#### Brain

Of participants with PCOS and controls, 12.9% and 9.3%, respectively, underwent brain MRI. No significant differences existed between groups with respect to any parameter, including grey and white matter volumes or hyperintensities.

### Subgroup Analysis According to PCOS Phenotype

Of the 6025 participants originally included, 142 participants were identified as having hyperandrogenic PCOS, 617 as having normoandrogenic PCOS, and 3775 as controls (Fig. 1). Baseline demographics are presented in Supplemental Table 12 (22). All CPHM results for subgroup analysis primary outcomes can be found in Table 4, while forest plots demonstrating respective HRs are presented in Supplemental Fig. 6 (22). E-values are presented in Supplemental Table 13 (22).

**Table 4.** Number, crude rates, and associated HRs for incident metabolic, cardiovascular, cancer, and dementia events in women with PCOS stratified by phenotype during subgroup analysis

Outcome	Group	No.	Events	Mean follow up, years (SD)	Incidence rate per 1000 person	Univariate model	Multivariable model 1	Multivariable model 2	Multivariable model 3	Multivariable model 4
Type 2 diabetes, HR (95% CI)										
Type 2 diabetes	Controls	3625	172	13.7 (2.1)	47.5	1.00	1.00	1.00	1.00	1.00
	NA-PCOS	587	35	13.6 (2.2)	59.6	1.27 (0.88-1.82)	1.31 (0.89-1.91)	1.27 (0.87-1.87)	1.19 (0.81-1.75)	1.35 (0.91-2.00)
	HA-PCOS	121	16	13.0 (3.0)	132.2	**2.96** (**1.78-4.95)**	**2.78 (1.61-4.82)**	**2.37 (1.37-4.11)**	**1.91 (1.10-3.32)**	1.39 (0.79-2.44)
Cardiovascular disease, HR (95% CI)										
All-cause CVD	Controls	3677	183	13.9 (1.7)	49.8	1.00	1.00	1.00	1.00	1.00
	NA-PCOS	599	48	13.6 (2.0)	80.1	**1.64** (**1.20-2.56)**	**1.79 (1.29-2.49)**	**1.77 (1.27-2.45)**	**1.82 (1.29-2.56)**	**1.90 (1.35-2.69)**
	HA-PCOS	134	12	13.6 (2.1)	89.6	**1.86** (**1.04-3.34)**	**2.45 (1.36-4.43)**	**2.23 (1.23-4.04)**	1.45 (0.77-2.75)	1.31 (0.69-2.49)
Cancer, HR (95% CI)										
All-cause Hormone-dependent cancer	Controls	3675	168	13.7 (2.1)	45.7	1.00	1.00	1.00	1.00	1.00
	NA-PCOS	598	37	13.5 (2.2)	61.9	1.36 (0.95-1.94)	1.34 (0.91-1.95)	1.33 (0.91-1.95)	1.31 (0.89-1.94)	1.33 (0.90-1.97)
	HA-PCOS	139	8	13.5 (2.4)	57.6	1.28 (0.63 -2.56)	1.13 (0.50-2.57)	1.17 (0.51-2.65)	0.77 (0.28-2.09)	0.69 (0.25-1.87)
Dementia, HR (95% CI)										
Dementia	Controls	3774	19	13.9 (1.6)	5.0	1.00	1.00	1.00	1.00	1.00
	NA-PCOS	617	4	13.9 (1.4)	6.5	1.30 (0.44-3.81)	1.63 (0.54-4.89)	1.62 (0.53-4.92)	1.71 (0.56-5.26)	1.71 (0.56-5.26)
	HA-PCOS	142	0	13.7 (2.0)	0	0.05 (0.00- 2404.98)	0.00 (0.00-NA)	0.00 (0.00-NA)	0.00 (0.00-NA)	0.00 (0.00-NA)

Model 1: age, Index of Multiple Deprivation, ethnicity; model 2: age, Index of Multiple Deprivation, ethnicity, smoking, alcohol; model 3: age, Index of Multiple Deprivation, ethnicity, smoking, alcohol, dyslipidemia, hypertension, cardiovascular disease, and/or glycated hemoglobin (dependent upon outcome); model 4: body mass index. Results in bold carry significance.

Abbreviations: CI, confidence interval; HA, hyperandrogenic; HR, hazard ratio; NA, normoandrogenic; PCOS, polycystic ovary syndrome.

#### T2D

The T2D incidence rate per 1000 person-years was higher in those with hyperandrogenic PCOS compared to normoandrogenic PCOS and controls (132.2 vs 59.6 vs 47.5). Univariate analysis revealed a significant association between participants with hyperandrogenic PCOS and T2D incidence (HR 2.96; 95% CI, 1.78-4.95), which disappeared after full adjustment.

#### CVD

The all-cause CVD incidence rate per 1000 person-years was higher in those with hyperandrogenic PCOS compared to normoandrogenic PCOS and controls (89.6 vs 80.1 vs 49.8). Univariate analysis revealed significant associations between PCOS and all-cause CVD incidence in those with hyperandrogenic (HR 1.86; 95% CI, 1.04-3.34) and normoandrogenic (HR 1.64; 95% CI, 1.20-2.56) phenotypes. The significant association remained after full multivariable adjustment only in those with normoandrogenic PCOS (HR 1.90; 95% CI, 1.35-2.69).

#### Cancer

The all-cause hormone-dependent cancer incidence rate per 1000 person-years was highest in those with normoandrogenic PCOS compared to hyperandrogenic PCOS and controls (61.9 vs 57.6 vs 45.7). Univariate analysis revealed no significant associations between PCOS phenotypes and controls, which remained after full multivariable adjustment.

#### Dementia

The incidence rate of dementia per 1000 person-years was highest in those with normoandrogenic PCOS compared to hyperandrogenic PCOS and controls (6.5 vs 0.0 vs 5.0). Univariate analysis revealed no significant associations between PCOS phenotypes and controls, which remained after full multivariable adjustment.

#### Multiorgan imaging

Of participants with hyperandrogenic PCOS, 9.9%, 8.5%, and 9.9% had undergone abdominal, cardiac, and brain MRI, respectively, while 10.9%, 10.5%, and 12.6% of participants with normoandrogenic PCOS and 8.1%, 8.0%, and 9.7% of controls had undergone abdominal, cardiac, and brain MRI, respectively. Participants with hyperandrogenic PCOS had significantly higher PDFF (9.0% vs 6.0% vs 5.2%; *P* < .01) and liver cT1 (776.3 ms vs 707.7 ms vs 701.9 ms; *P* < .01) than participants with normoandrogenic PCOS and controls after adjusting for BMI [Supplemental Fig. 5 (22)]. No significant differences existed between those with normoandrogenic PCOS and controls [Supplemental Table 14 (22)].

## Discussion

In this large, prospective analysis of UKBB data, we found that a previous PCOS diagnosis significantly increased prevalent and incident metabolic (T2D and MASLD) and CVD risk compared to age- and BMI-matched controls, although risk of hormone-dependent cancer and dementia was comparable to the general population. Additionally, abdominal MRI demonstrates a higher prevalence of hepatic steatosis and greater hepatic fibroinflammation in participants with PCOS. Differences were not explained by such traditional lifestyle or demographic risk factors as smoking, alcohol, sedentariness, or ethnicity, with detailed baseline biochemical and reproductive history characterization of our participants providing validity of the previous PCOS diagnosis. When subgrouped by PCOS phenotype, our results suggest hyperandrogenism may contribute to incident metabolic disease, perhaps mediated through liver fat/fibroinflammation, while conferring protection against CVD, when compared to normoandrogenic PCOS. We provide the most detailed phenotypic characterization including assessment of lifestyle factors and multiorgan MRI to provide mechanistic insight into the higher rates of long-term complications in these women.

### Metabolic Disease

The results of our study align with existing literature assessing the association between PCOS and incident T2D (8-10). The smaller effect size in our study may relate to our participants being postmenopausal and already having established T2D, whereas other studies analyzed premenopausal patients (8-10). In fact, prevalence of T2D in our PCOS participants was 7.3%, whereas previous literature has reported a prevalence as low as 1.9% (9). Importantly, we propose the risk of T2D in people with PCOS may be related to liver health and the greater proportion with MASLD. Both liver fat (26) and fibroinflammation (27) are associated with T2D, perhaps through genetic predisposition (28). In our cohort, participants with PCOS more commonly had hepatic steatosis and hepatic fibroinflammation (evidenced by the liver fibrosis noninvasive tests and abdominal imaging findings), consistent with previous work (3).

### CVD

Although heterogenous methodology and underpowered sample sizes cloud the results of prospectively designed research (5, 6, 11, 20), larger retrospective analyses align with our work in demonstrating an increased risk of CVD in patients with PCOS (7, 12). This may be particularly true for acute coronary syndrome and heart failure, but not stroke, as with our results (7). Traditional cardiovascular risk factors, such as obesity and insulin resistance, likely contribute to CVD pathophysiology (18); however, participants in our study were matched for BMI, with comparable ethnic composition and sedentariness, and lesser smoking and alcohol consumption. These conclusions are concordant with previous studies suggesting higher rates of incident CVD in people with PCOS are independent of traditional risk factors (11, 12).

### Cancer and Dementia

The association between PCOS and other long-term health consequences, such as hormone-dependent cancer and dementia, is less clear. PCOS patients may be at increased risk of endometrial and ovarian cancer, but their risk of breast and colorectal cancer is akin to the general population (15-17). Although our study found no significant associations between PCOS and any cancer, we may have been underpowered to detect differences here, as the total number of events for endometrial and ovarian cancer in the total cohort was 6 and 33, respectively. Similarly, we did not find an association between PCOS and dementia or differences in brain matter volume between the groups of postmenopausal women. Previous brain MRI analysis in younger people with PCOS found altered white matter microstructure, with subtly compromised cognitive performance (13).

### Phenotyping by Androgens

We confirm our previous work with evidence that hyperandrogenic PCOS represents a distinct metabolic phenotype, characterized by hepatic steatosis and fibroinflammation, independent of BMI and adipose tissue volume, suggesting later stages of MASLD are present in these patients (3). The mechanisms underlying this are dichotomous and exist within a sexual dimorphism, but, in females, androgens reduce liver fat export, by binding to hepatic low-density lipoprotein receptors, which impairs very low density lipoprotein transport of hepatic triglycerides (29), and/or fatty acid oxidation, exacerbating fibroinflammation through overexpression of interleukins and tumor necrosis factors (30).

As well as the association between hepatic fibroinflammation and T2D (26, 27), elevated liver cT1 may also predict cardiovascular events (31). Despite participants with PCOS and hyperandrogenism having more fibroinflammation, they were at no increased risk of CVD, whereas their normoandrogenic counterparts were, suggesting cardioprotective properties of postmenopausal hyperandrogenism. A recent review highlights that these findings have been consistently replicated in existing literature, with postmenopausal hyperandrogenic PCOS patients having a similar overall risk of CVD and coronary artery disease compared to the general population, although underpinning mechanisms warrant further investigation (32).

### Future Directions and Clinical Implications

More careful characterization of PCOS phenotype, specifically addressing clinical and biochemical androgen status, with tailored surveillance based on the phenotype-specific differential risk is needed. The Rotterdam criteria does not mandate FAI calculation for all new PCOS diagnoses, and most guidelines do not consider routine screening of MASLD (33). Based on our findings, liver disease is a relevant consideration in patients with hyperandrogenic PCOS. Fibrosis index-4 testing may provide a useful screening tool in addition to transient elastography (Fibroscan) for which controlled attenuation parameters and liver stiffness measurements have been found to correlate well with MRI-determined PDFF and cT1, respectively (34). Finally, an emphasis on randomized clinical trials and real-world data is needed to investigate the most cost-effective methods for the primary and secondary prevention of cardiometabolic disease in people with PCOS. Novel incretin-based therapy, such as mono, dual, and triple receptor agonists, may have merit in reducing the burden of steatotic liver disease (35), specifically those agents incorporating glucagon agonism, such as retatrutide, due to the direct hepatic activity of glucagon (36). This is imperative to address now, given the likely tsunami of major metabolic liver-related disease/cirrhosis in the future.

### Strengths and Limitations

Our study has many strengths. First, the UKBB provides a large, prospective, general population-based cohort, representative of the UK, while enabling us to perform extensive phenotypic characterization of participants with lifestyle, biochemical, and imaging data to provide mechanistic insight into their long-term health outcomes. This characterization allows demonstration of differences in outcomes as being independent of traditional demographic/lifestyle risk factors. Moreover, the long follow-up in older people with PCOS enabled the identification of incident disease, which was less likely to have occurred in younger people.

However, we do acknowledge limitations. First, the absence of transvaginal ultrasound imaging data means it is possible that some controls may have had undiagnosed PCOS. We attempted to overcome this by identifying previously undiagnosed cases of PCOS through biochemical evaluation and codes for hyperandrogenism and a/oligomenorrhoea, although we acknowledge that there may have been participants with ovarian morphology characteristic of PCOS on pelvic ultrasonography (either 12 or more follicles measuring 2-9 mm in diameter and/or an increased ovarian volume >10 cm^3^), which if combined with 1 or more features of hyperandrogenism or a/oligomenorrhoea would fulfill the diagnostic criteria of PCOS. However, the lack of this imaging data meant we could not confirm whether this was the case. Moreover, given that the participants in the current study were largely peri- or postmenopausal, and therefore their androgen, or indeed other sex steroid, profiles may have changed with time, it is possible that some of the controls may also have had biochemical features of hyperandrogenism during their premenopausal years that had subsequently resolved. This again may have led to some diagnostic misclassifications. Additionally, the UKBB uses electronic health records, leading to a potential for lack of data completeness as well as residual confounding and reporting bias. For example, metformin can, of course, influence subsequent development of T2D in those at risk as shown in the Diabetes Prevention Program (37). As we highlight from our results, patients with PCOS are at significantly greater risk of incident T2D. It is likely that this risk of incident T2D would be diminished by the prescription of metformin, and thus we might expect that rates of T2D may be slightly higher in participants with PCOS who were not prescribed metformin. This would mean that our effect size may be a conservative estimate that underestimates the true risk that would be observed if none of the PCOS cohort was taking metformin. However, we have not included metformin in the covariate model, considering that data regarding the initiation date of the drug, and the compliance with the medication following initiation, was not fully available, and therefore this would render the multivariable analysis less robust. Similarly, for antiandrogenic medication or oral contraceptives, we have poor data regarding start date or adherence. The combined oral contraceptive pill tends to be prescribed for the regulation of menses but usually only until menopause, and, similarly, antiandrogens will be discontinued at a similar time. Participants in the UKBB are largely postmenopausal, and therefore prescription of these drugs will have been for a limited (and difficult to ascertain) time frame only. However, we are not aware of any evidence to suggest that the combined oral contraceptive pill modifies the risk of CVD or T2D either negatively or positively (38). We did perform sensitivity analyses by generating E-values to demonstrate the strength of association that an unmeasured confounding variable would need to have to explain away any association between PCOS and the outcome of interest. Moreover, ∼85% of participants were of White ethnicity, meaning this data is most relevant to this population, and further evaluation is required to confirm findings in other ethnicities, while coding for participants who have undergone sex transitioning is limited. To the best of our ability, we tried to identify any control participant with discordant sexual coding using ICD-10 codes for transsexualism, although we found none. Finally, we used complete case analysis during our survival analysis, which may have led to underpowering of some study findings, particularly in relation to hormone-dependent cancers, dementia, and phenotype analysis.

## Conclusion

In summary, we highlight that people with PCOS are at an increased risk of metabolic disease and CVD, with a potential divergence of risk according to PCOS phenotype. We note hyperandrogenism may confer opposing metabolic and cardiovascular effects, with a potentially amplified risk of MASLD despite a lower risk of CVD. These results suggest more precise phenotype classification may benefit ongoing risk stratification for patients with PCOS.

## Data Availability

The data that support the findings of this study are available from the UK Biobank (https://www.ukbiobank.ac.uk/).

## References

[1] Deswal R, Narwal V, Dang A, Pundir CS. The prevalence of polycystic ovary syndrome: a brief systematic review. J Hum Reprod Sci. 2020;13(4):261‐271. 33627974 10.4103/jhrs.JHRS_95_18 PMC7879843

[2] Rotterdam ESHRE/ASRM-Sponsored PCOS consensus workshop group . Revised 2003 consensus on diagnostic criteria and long-term health risks related to polycystic ovary syndrome (PCOS). Hum Reprod. 2004;19(1):41‐47. 14688154 10.1093/humrep/deh098

[3] Jones H, Sprung VS, Pugh CJA, et al Polycystic ovary syndrome with hyperandrogenism is characterized by an increased risk of hepatic steatosis compared to nonhyperandrogenic PCOS phenotypes and healthy controls, independent of obesity and insulin resistance. J Clin Endocrinol Metab. 2012;97(10):3709‐3716. 22837189 10.1210/jc.2012-1382

[4] Dobbie LJ, Pittam B, Zhao SS, et al Childhood, adolescent, and adulthood adiposity are associated with risk of PCOS: a Mendelian randomization study with meta-analysis. Hum Reprod. 2023;38(6):1168‐1182. 37015099 10.1093/humrep/dead053 PMC10233304

[5] Merz CN, Shaw LJ, Azziz R, et al Cardiovascular disease and 10-year mortality in postmenopausal women with clinical features of polycystic ovary syndrome. J Womens Health (Larchmt). 2016;25(9):875‐881. 27267867 10.1089/jwh.2015.5441 PMC5311460

[6] Schmidt J, Landin-Wilhelmsen K, Brännström M, Dahlgren E. Cardiovascular disease and risk factors in PCOS women of postmenopausal age: a 21-year controlled follow-up study. J Clin Endocrinol Metab. 2011;96(12):3794‐3803. 21956415 10.1210/jc.2011-1677

[7] Glintborg D, Rubin KH, Nybo M, Abrahamsen B, Andersen M. Cardiovascular disease in a nationwide population of danish women with polycystic ovary syndrome. Cardiovasc Diabetol. 2018;17(1):37. 29519249 10.1186/s12933-018-0680-5 PMC5844097

[8] Ryu KJ, Kim MS, Kim HK, et al Risk of type 2 diabetes is increased in nonobese women with polycystic ovary syndrome: the national health insurance service-national sample cohort study. Fertil Steril. 2021;115(6):1569‐1575. 33509630 10.1016/j.fertnstert.2020.12.018

[9] Persson S, Elenis E, Turkmen S, Kramer MS, Yong EL, Poromaa IS. Higher risk of type 2 diabetes in women with hyperandrogenic polycystic ovary syndrome. Fertil Steril. 2021;116(3):862‐871. 34053678 10.1016/j.fertnstert.2021.04.018

[10] Liao WT, Huang JY, Lee MT, Yang YC, Wu CC. Higher risk of type 2 diabetes in young women with polycystic ovary syndrome: a 10-year retrospective cohort study. World J Diabetes. 2022;13(3):240‐250. 35432752 10.4239/wjd.v13.i3.240 PMC8984565

[11] Ollila M-M, Arffman RK, Korhonen E, et al Women with PCOS have an increased risk for cardiovascular disease regardless of diagnostic criteria—a prospective population-based cohort study. Eur J Endocrinol. 2023;189(1):96‐105. 37436934 10.1093/ejendo/lvad077

[12] Berni TR, Morgan CL, Rees DA. Women with polycystic ovary syndrome have an increased risk of Major cardiovascular events: a population study. The Journal of Clinical Endocrinology & Metabolism. 2021;106(9):e3369‐e3380. 34061968 10.1210/clinem/dgab392 PMC8372630

[13] Rees DA, Udiawar M, Berlot R, Jones DK, O'Sullivan MJ. White matter microstructure and cognitive function in young women with polycystic ovary syndrome. J Clin Endocrinol Metab. 2016;101(1):314‐323. 26574952 10.1210/jc.2015-2318 PMC4701841

[14] Vgontzas AN, Legro RS, Bixler EO, Grayev A, Kales A, Chrousos GP. Polycystic ovary syndrome is associated with obstructive sleep apnea and daytime sleepiness: role of insulin resistance1. J Clin Endocrinol Metab. 2001;86(2):517‐520. 11158002 10.1210/jcem.86.2.7185

[15] Frandsen CLB, Svendsen PF, Nøhr B, et al Risk of epithelial ovarian tumors among women with polycystic ovary syndrome: a nationwide population-based cohort study. Int J Cancer. 2023;153(5):958‐968. 37357906 10.1002/ijc.34574

[16] Gottschau M, Kjaer SK, Jensen A, Munk C, Mellemkjaer L. Risk of cancer among women with polycystic ovary syndrome: a danish cohort study. Gynecol Oncol. 2015;136(1):99‐103. 25451694 10.1016/j.ygyno.2014.11.012

[17] Ding DC, Chen W, Wang JH, Lin SZ. Association between polycystic ovarian syndrome and endometrial, ovarian, and breast cancer: a population-based cohort study in Taiwan. Medicine (Baltimore). 2018;97(39):e12608. 30278576 10.1097/MD.0000000000012608 PMC6181615

[18] Glueck CJ, Dharashivkar S, Wang P, et al Obesity and extreme obesity, manifest by ages 20-24 years, continuing through 32-41 years in women, should alert physicians to the diagnostic likelihood of polycystic ovary syndrome as a reversible underlying endocrinopathy. Eur J Obstet Gynecol Reprod Biol. 2005;122(2):206‐212. 16219521 10.1016/j.ejogrb.2005.03.010

[19] Cassar S, Misso ML, Hopkins WG, Shaw CS, Teede HJ, Stepto NK. Insulin resistance in polycystic ovary syndrome: a systematic review and meta-analysis of euglycaemic-hyperinsulinaemic clamp studies. Hum Reprod. 2016;31(11):2619‐2631. 27907900 10.1093/humrep/dew243

[20] Meun C, Franco OH, Dhana K, et al High androgens in postmenopausal women and the risk for atherosclerosis and cardiovascular disease: the rotterdam study. J Clin Endocrinol Metab. 2018;103(4):1622‐1630. 29408955 10.1210/jc.2017-02421

[21] UK Biobank . About us UK Biobank 2023. Accessed July 18, 2024. https://www.ukbiobank.ac.uk/learn-moreabout-uk-biobank/about-us

[22] Henney A, Gillespie C, Lai, J, et al Supplementary Material. 2024.

[23] Lee JH, Kim D, Kim HJ, et al Hepatic steatosis index: a simple screening tool reflecting nonalcoholic fatty liver disease. Dig Liver Dis. 2010;42(7):503‐508. 19766548 10.1016/j.dld.2009.08.002

[24] Ożga K, Krzyczkowska-Sendrakowska M, Hubalewska-Dydejczyk A, et al The value of the free androgen index depends on the phenotype of polycystic ovary syndrome—a single-centre experience. Endokrynol Pol. 2019;70(4):330‐335. 30938834 10.5603/EP.a2019.0017

[25] VanderWeele TJ, Ding P. Sensitivity analysis in observational research: introducing the E-value. Ann Intern Med. 2017;167(4):268‐274. 28693043 10.7326/M16-2607

[26] Martin S, Sorokin EP, Thomas EL, et al Estimating the effect of liver and pancreas volume and fat content on risk of diabetes: a Mendelian randomization study. Diabetes Care. 2022;45(2):460‐468. 34983059 10.2337/dc21-1262

[27] Waddell T, Bagur A, Cunha D, et al Greater ectopic fat deposition and liver fibroinflammation and lower skeletal muscle mass in people with type 2 diabetes. Obesity (Silver Spring). 2022;30(6):1231‐1238. 35475573 10.1002/oby.23425 PMC9321120

[28] Yaghootkar H, Lotta LA, Tyrrell J, et al Genetic evidence for a link between favorable adiposity and lower risk of type 2 diabetes, hypertension, and heart disease. Diabetes. 2016;65(8):2448‐2460. 27207519 10.2337/db15-1671 PMC5386140

[29] Baranova A, Tran TP, Afendy A, et al Molecular signature of adipose tissue in patients with both non-alcoholic fatty liver disease (NAFLD) and polycystic ovarian syndrome (PCOS). J Transl Med. 2013;11(1):133. 23721173 10.1186/1479-5876-11-133 PMC3681627

[30] Zhang Y, Meng F, Sun X, et al Hyperandrogenism and insulin resistance contribute to hepatic steatosis and inflammation in female rat liver. Oncotarget. 2018;9(26):18180‐18197. 29719598 10.18632/oncotarget.24477 PMC5915065

[31] Roca-Fernandez A, Banerjee R, Thomaides-Brears H, et al Liver disease is a significant risk factor for cardiovascular outcomes—a UK biobank study. J Hepatol. 2023;79(5):1085‐1095. 37348789 10.1016/j.jhep.2023.05.046

[32] Hirschberg AL . Hyperandrogenism and cardiometabolic risk in Pre- and postmenopausal women-what is the evidence? J Clin Endocrinol Metab. 2023;108(5):1243‐1253. 37886900 10.1210/clinem/dgad590 PMC11031217

[33] Legro RS, Arslanian SA, Ehrmann DA, et al Diagnosis and treatment of polycystic ovary syndrome: an endocrine society clinical practice guideline. J Clin Endocrinol Metab. 2013;98(12):4565‐4592. 24151290 10.1210/jc.2013-2350 PMC5399492

[34] Oeda S, Tanaka K, Oshima A, Matsumoto Y, Sueoka E, Takahashi H. Diagnostic accuracy of FibroScan and factors affecting measurements. Diagnostics (Basel). 2020;10(11):940. 33198092 10.3390/diagnostics10110940 PMC7696616

[35] Mantovani A, Petracca G, Beatrice G, Csermely A, Lonardo A, Targher G. Glucagon-Like peptide-1 receptor agonists for treatment of nonalcoholic fatty liver disease and nonalcoholic steatohepatitis: an updated meta-analysis of randomized controlled trials. Metabolites. 2021;11(2):73. 33513761 10.3390/metabo11020073 PMC7911747

[36] Jastreboff AM, Kaplan LM, Frías JP, et al Triple–hormone-receptor agonist retatrutide for obesity—a phase 2 trial. N Engl J Med. 2023;389(6):514‐526. 37366315 10.1056/NEJMoa2301972

[37] Knowler WC, Barrett-Connor E, Fowler SE, et al Reduction in the incidence of type 2 diabetes with lifestyle intervention or metformin. N Engl J Med. 2002;346(6):393‐403. 11832527 10.1056/NEJMoa012512 PMC1370926

[38] Vrbíková J, Cibula D. Combined oral contraceptives in the treatment of polycystic ovary syndrome. Hum Reprod Update. 2005; 11(3):277‐291. 15790599 10.1093/humupd/dmi005

