# Correction to: “Risk of Type 2 Diabetes, MASLD and Cardiovascular Disease in People Living With Polycystic Ovary Syndrome”

**DOI:** 10.1210/clinem/dgaf643

**Published:** 2025-12-04

In the above-named article by Henney AE, Gillespie CS, Lai JYM, Schofield P, Riley DR, Caleyachetty R, Barber TM, Miras AD, Dobbie LJ, Hughes DM, Alam U, Hydes TJ, and Cuthbertson DJ (*J Clin Endocrinol Metab*. 2025, 110(5): 1235-1246; doi: 10.1210/clinem/dgae481) there was an error in the authorship roster.

In the original article, Conor S. Gillespie name was incorrectly published as “Conor S. Gillespiec.” In the updated article, Conor S. Gillespie’s name has been listed correctly.

The publisher acknowledges this error.

The article has been corrected online.

doi: 10.1210/clinem/dgae481

